# After Six Years of Dentistry

**Published:** 1903-10

**Authors:** Charles E. Parkhurst

**Affiliations:** Somerville, Mass.


### AFTER SIX YEARS OF DENTISTRY.[1]

BY CHARLES E. PARKHURST, A.B., D.M.D., SOMERVILLE, MASS.

In a retrospect of my experiences of a brief period as a dentist I am confronted with the fact that most of my methods of work are entirely different from those learned at school and constituting my armament when migrating therefrom. Any wide-awake man who practises our profession to-day must be impressed with the reality that modern dentistry is making more rapid advances along all lines than any other profession. By modern dentistry I mean that advocated and practised by the most progressive men. There is a large percentage who reached the acme of their career long ago and are content to practise the modes of their fathers.

I am intensely proud of my occupation, and while there have been times when I have coveted the medical training, I have come to think that the successful all-around dentist has about all he can attend to to acquit himself creditably in his own line of work. Our school courses are of great help, but, after all, success lies in the man himself, and not in the school product. The dental graduate as he emerges from school is just on the stepping-stones of his activity. With the instruction received from school, that which the graduate needs more than all else is experience and a superabundance of good sense or judgment,—"horse sense," if you please,—the power of discrimination or applicability of the proper method of procedure.

Thrown upon one's own resources, he meets with success who possesses the faculty to diagnose properly a case and has at his disposal various ways of accomplishing his end. That man makes no marked success who considers that the knowledge absorbed at school fits and equips him for future time.

Dentistry is changing so rapidly to-day that one must almost lie awake nights to keep abreast with the presentation of new and the overthrow of well-established ideas.

As a continuation of one's dental education post-graduate courses are available, and should be grasped by all. These are largely in the shape of dental meetings and magazines, and he who does not profit by these helps cannot expect to reach the front.

---

[1] **Read before the Harvard Dental Alumni Association, June 22, 1903.**

Are we readers of the best dental magazines, and do we read them in their entirety? Do we omit some articles because too dry and scientific? If such is the case, persistency should be maintained until a taste is cultivated for these. The National, State, and other dental meetings of the year are invaluable as furnishing yearly new thoughts and methods and affording a periodical stimulus to other work. Having endeavored in every way to continue the pursuit of dental education, and in so doing, by absorbing new ideas and in following out certain ways which have been practised, the writer would suggest some helpful methods adopted to-day as a result of a limited experience. All this by way of introduction.

In operative dentistry I have come to think that the fewer the instruments the better. Having been taught by some to use the rubber in most all cases, I now find it necessary to use it only in connection with certain gold fillings. I do not deem it essential to ligate always the teeth involved, and especially the double ligature. Neither do I see the appropriateness of the ligature instrument suggested in my school days. That might answer for teeth with pyorrhœa pockets, but not for the closely hugging gums usually encountered. The Libby flat burnisher serves my purpose to best advantage to carry silk under the gum. Cotton rolls made by twisting a tuft of cotton on a dampened pointed orange-wood stick used in connection with a suitable clamp for retention, together with a matrix when indicated, permit to do all treating and filling, gold generally excepted. With frequent changes of these rolls, the field of operation can be kept dry as long as desired.

The treatment of teeth in all its phases is the most critical and exhausting, and requires as much skill as does any dental operation. No work is so shiftlessly performed by practitioners as a whole. There is no short cut to successful treatment. It is frequently a question of hours to accomplish, and just and commensurate remuneration should be received. I do not believe in any mummifying method. To mummify is to shrink or contract with the consequent space between the shrivelled remnant of pulp and tooth walls, which space serves as a receptacle for serum and gases from the apical region. Well-known results follow this condition. Mummifying may do for subjects which are to remain in non-active surroundings, but not for teeth with vital surroundings. Undoubtedly this method does work well for a time, but I cannot but think it has a time limit. A clear field, clean broaches, thorough mechanical cleansing and removal of all remnants of pulp or débris from all canals as far as possible, thorough disinfection, and stopping of canals with antiseptic root-filling has met with reward as to permanency of results. In pulpless teeth, with the pulp-chamber well opened, not access around the corner, the location of canals in view, those not readily cleansed by broaches and neutralized dioxide, can be entered generally by sulphuric acid and a smooth broach if patient and persistent, the sulphuric acid to be neutralized after using with bicarbonate of soda, although I think that the lime-salts of the tooth neutralize and limit its action to a great extent. Suitable smooth broaches for the work can be obtained for five dollars per gross. Incidentally I have found that economy should not be exercised in too long retention of broaches. Discard and take a new one before it is time for the old one to break. Broaches are dipped in carbolic acid and passed through the flame before using.

As stated before, peroxide is my regular canal cleanser. I like it because I can see it work, and it throws out of the canals the débris by its effervescence. Canal dressings are eucalyptol, beechwood, creosote, and formalin,—eucalyptol, or eucalyptol and formalin, in anterior teeth; the same, or creosote, with or without formalin, in posterior teeth. And in stubborn cases forty per cent. alone has proved efficient. When a pulpless tooth is first opened and cleansed, it is stopped with cotton, then cotton and varnish, and finally gutta-percha, or temporary stopping.

I wish to say that, contrary to the practice of many, I use whatever drug is most efficient for the case in hand, iodoform not excepted. I do not consider the fastidious olfactory sensation of my patients if I am using, to my mind, the best means to an end to effect a permanent and speedy cure. I do not believe that my practice lessens materially because of this procedure.

Chronic abscesses of anterior teeth with fistula are treated by cleansing with peroxide through the teeth, and that followed by chloropercha. If the case fails to heal, the opening is packed two or three times with twine dipped in eucalyptol to enlarge. Then, with a cross-cut bur, the end of the root or neighboring bony tissue is removed to a slight extent and the healing process generally takes place. I do not see the need in so many cases of using a large bone trephine or bur, and removing as much sound or diseased tissue, possibly the apex of a neighboring tooth, and subjecting the patient to considerable unnecessary pain. So much for the pulpless teeth.

Now to those with pulps or partial pulps. When to devitalize. If the cavity is presented and the pulp is exposed, devitalize at once. I do not cap. If I have capped, I uncap later. If tooth has ached to any extent, especially at night, or except from local irritation, I devitalize. The pressure method, with unvulcanized rubber or yellow wax, large instrument as plunger, crystal of cocaine with drop of alcohol or chloroform is used almost entirely to anæsthetize preparatory to the removal of pulp. By this same method an equal mixture of carbolic acid and chloroform instead of cocaine works quite as satisfactorily. One is better in some cases than the other. That annoying particle of sensation at the apex of the root can ordinarily be removed by pumping carbolic acid and chloroform into the root with a smooth broach. A new barbed broach is always used to remove a pulp. Applications of arsenic or fibre are seldom or hardly ever used. Having tried all suggested methods for painless results, the pain invariably does follow an application in molar teeth. An application of arsenic or fibre as an obtundent for a short time, followed by cocaine and pressure or cataphoresis with cocaine to obtain clean-cut exposure, then by cocaine with pressure, has met with good results. Except when the pulp is congested, in which case most any method is painful, the pressure method seems to be ideal. Pain in this process results often for the want of an actual exposure. The mere film of dentine over the pulp, although it is flexible, is a barrier to the ready transfer of the cocaine. If the pulp can be made to bleed a little, the method will work to perfection.

FILLING ROOTS.

When the pulp is detached entire and but little or no hemorrhage follows its removal, fill the roots immediately. My experience is that immediate canal filling is seldom advisable in molar teeth, as one cannot remove all the remnants of the pulp at once. Those minute canals are not so easily untenanted and require much time to cleanse and disinfect. If a canal is so fine and tortuous that I am conscious that I do not penetrate to its end, even with sulphuric acid, I rely upon repeated dressings to disinfect and saturate the root previous to its filling. The root-filling consists of pink gutta-percha, because of its color, which can be traced in drilling for a post, or in case of removal. This is dissolved in chloroform, cajeput-eucalyptus, with the addition of hydronaphtol, oil of cassia, and iodoform. This is always heated in a water-bath just previous to using, to render it of a creamy consistency. Peroxide is used first, then alcohol, and finally eucalyptol, after which the canal is dried with hot air. The root-filling is pumped into the canals with a smooth broach, not a barbed one with a shred of cotton on the end, as this acts too much like a piston and is too liable to force the chloropercha through the apex. The eucalyptol left in the canal acts as a lubricant and helps the chloropercha to follow to the apex. When reasonably sure that the canal is filled, a point of gutta-percha of the appropriate size is used carefully as a piston, and then left to furnish a hard centre of the root-filling. These gutta-percha points, by the way, can be made very nicely by rolling out small pieces into long cones pointed at either end on a heated tile, and when cold cutting in the centre.

I wish the tooth or surrounding tissue to present or have presented no soreness for some days previous to the root-filling. I desire to let not more than a week elapse between the changing of the dressing and the root-filling. In most all cases of trouble with "teeth said to have been treated," the cause of the soreness or pain is evident upon opening into the tooth. Fillings over uncleansed pulp-chamber, uncleansed canal or canals, unsought canals, cotton in roots, are among the disclosures met daily. Cotton left in canals in this day is work open to the highest criticism, and teeth which do not evince the most careful work in their treatment to-day are signs of a shiftless and unscrupulous workman, who, from an ethical or non-ethical stand-point, is open to censure.

### CHILDREN'S TEETH.

These teeth, as well as their possessors, are frequently hard to manage. Crown cavities, after excavation, are coated with nitrate of silver and filled with amalgam; proximal cavities are treated in the same manner and filled with cement and amalgam. If the pulp is exposed, anæsthetize with carbolic acid and chloroform, open up, remove the pulp with a large spoon, check hemorrhage, and fill with chloropercha, saturating a tuft of cotton with this and leaving it in the tooth, covering this with gutta-percha, and completing with cement and amalgam. If the child is young and restless, merely coat with nitrate of silver, or use Ames's black oxyphosphate of copper. The cavity need not be wholly free from moisture in using this. I have had no success with capping children's teeth. I do not use cement or gutta-percha as a filling-material for these teeth. If the tooth is pulpless, and there is a fistula established, peroxide and chloropercha forced through the fistula is the remedy. The engine is used early with children to dispel the fear of this contrivance.

### CAPPING FOR OTHER TEETH (WHEN INDICATED).

It is used in cases where the cavity is near the pulp, but not an exposure. It is a precautionary or decay-restraining step, if such is possible. The capping consists of jodoformagen and iodoform powder, equal parts, mixed with oil of cloves as the liquid. Some of this powder and liquid is placed upon a tile, a small thin spatula heated over the burner, the mixture made and applied to the floor of the cavity with a small piece of cotton held in pliers. It will harden quickly with hot air. In case the floor of the cavity is thin, a coating of cement should be flowed over before regular filling.

### FILLING-MATERIALS.

Gold and cement, amalgam and cement, porcelain, oxyphosphate of copper, very rarely gutta-percha, no cement. More and more, porcelain, except gold, appeals to me to be the material to be inserted when the cavity is accessible and of a moderate size. If not accessible, a plastic is preferable. For the past year I have used almost exclusively porcelain, except gold and amalgam in combination with cement, and have relied upon the cement to retain the filling. Practically no other means of retention is obtained; scarcely a groove or undercut; only a good depth to cavity is desired. I know that idealists depict cavities so shaped with broad cervical margins and self-retentive proximal walls that no grooves or undercuts are necessary, but the ordinary practitioner, methinks, to be on the safe side, will remove a little dentine along those same proximal walls, just as a precaution, as there is a desire that the filling "stay in," even if the tooth has to be cut overmuch to hold it. With the combination filling all decay is removed, and the edges smoothed and polished. The cavity is wiped with carbolic acid and alcohol, or carbolic alone, and dried with hot air. No lining is used, as I do not believe that the phosphoric acid in the cement has any decided action on the pulp. The matrix is used in most all cases of proximal cavities. I like Hood's matrix metal, cut to proper size and held in place at the cervix by a pointed toothpick, and about the circumference or margins of the tooth by an ivory clamp when cement or amalgam is used. With gold, a large piece of pink gutta-percha is softened and placed on either side of the tooth against the matrix metal, and held by the jaws of the clamp. The gutta-percha holds the metal in place and also prevents the clamp from pressing upon the gum, so that the clamp can be used to rest the finger upon in condensing the gold if desired.

### METHOD OF INSERTING AMALGAM AND CEMENT.

The amalgam is mixed, and in some cases, according to Dr. Clapp's method, a wall is built against the matrix and carefully pressed against the proximal margins; then some soft cement is placed with a pointed instrument into the pocket between the amalgam and the floor of the cavity; then amalgam is worked into the cement and the filling built up to contour. A broken-backed explorer with the point ground off makes a very useful instrument with which to convey the cement to the cavity. Another method is to place the cement in the cavity at first, then press the amalgam into the cement, letting the cement ooze out to margins, and while soft scrape off the surplus cement, adding the remainder of the amalgam to complete the contour. If doubtful of the margins, remove the clamp, turn back the matrix, and add amalgam to margins. In coronal cavities line with soft cement and work amalgam into same.

### GOLD FILLINGS.

No tooth is too soft or frail for gold and cement that one would restore with cement alone. Gold will protect and preserve the margins of a frail tooth better than any other material. With cement and gold, the floor of the cavity is covered with soft cement and immediately some De Trey's gold is worked into same, to cover all the cement. This is allowed to harden for about five minutes, and then condensed. The cervical and proximal walls are then cleansed and the matrix applied. Tin and gold, if not afraid of discoloration, or non-cohesive gold, is first used at the cervical margin, followed by some De Trey's gold. The proximal walls are then built up of Shumway's gold cylinders, because of their pliability or plasticity, sufficient force being exerted to head the matrix from the edge of tooth and leaving gold to be burnished down to margins in finishing after matrix is removed. The filling is finished by burnishing to margins with an Ivory point. In condensing the gold the Darby Perry No. 17 plugger, because of long shank, and one made with same point, but on an obtuse angle, are my most helpful pluggers. The Hopkins pluggers, if kept blue, make very good burnishing instruments. I use all plugger-points as much with a sliding motion over the surface of the gold, really burnishing it, as with the regular plugging action. No one variety of gold is used, but several kinds in the same filling if I see the appropriateness of them.

ADVANTAGES OF CEMENT IN COMBINATION WITH GOLD OR AMALGAM.

The pain of excavating is reduced to a minimum. The proximal walls are not weakened by grooves inviting redecay. Gold can be inserted anywhere in anterior teeth where one would use cement. No discoloration follows the use of amalgam with cement. A partial non-conducting lining of cement is used under all metal fillings, a broad floor of gold covering the whole surface of the cavity to which to build. No rocking of gold after starting, no building of cervix down to cutting edge before one is sure that filling is locked. The burden of filling is much lightened. The mistake in this method is to proceed with the filling before the cement is thoroughly set or crystallized, or to use it in corners of anterior teeth with no surrounding walls. It can be used in practically saucer-shaped cavities, but depth is necessary. In excessively sensitive posterior teeth with little wear oxyphosphate of copper serves a useful purpose. This is applicable to buccal cavities and fissures, and also to non-erupted wisdom-teeth. It can be applied in a creamy state, and will flow along the fissure, when a puff of hot air will cause it to set immediately. In case of removal or renewal the cavity will be found less sensitive.

Porcelain is used in anterior teeth when the patient is desirous of same and appreciates or can be made to appreciate the work. This is the nicest work possible if perfect, requires the most patience and skill, and is fraught with much disappointment, but there is a growing demand for it, and everybody should be able to do it well. The common excuse, "no call for such work," is hardly a safe or proper platform on which to stand. I have not reached that stage when I advise the removal of a perfect gold proximal filling in order to insert possibly, though not probably, so good a porcelain filling, because of the color of the gold. There are two exposures of an anterior filling,—the labial and palatal. A proximately perfect fitting labial surface of a porcelain filling with an ill-fitting palatal surface is not equal to a well-burnished tight-fitting gold filling upon both aspects. Tooth preservation is as important as æsthetic considerations. The wholesale removal of metal fillings and substitution of porcelain is open to criticism. I employ two different kinds of porcelain fillings. For anterior teeth generally the light-fusing bodies are used, and an endeavor is made to match the color as nearly as possible. For cervical cavities of bicuspid or molar teeth the color is not as important, and porcelain can be used, even if not a perfect match, as one would use cement. It can be used here because of ease of manipulation, shortness of operation, and avoidance of cervical clamp in case of gold filling and because of non-conducting qualities of body. In such class of cavities I use Jenkins's body and gold matrix. I confess to little success with this body because of inability to retain the color. With all care I find that it bleaches in melting. Where the color is not of prime importance there are two or three different methods for its use. An impression can be obtained with No. 30 gold with spunk and wet cotton ball, invested and filled with body, and baked, etched, and set.

### ANOTHER METHOD.

Gold is adapted to the cavity with spunk and a wet tuft of cotton, then softened pink base-plate gutta-percha is pressed into the cavity over the gold with the ball of thumb and burnishers and allowed to harden, maintaining the pressure to obtain well-defined margins. When hard, remove and invest; when investment is set, heat a little to soften the gutta-percha; remove, fill with body, and proceed as usual. The results are very satisfactory.

### A METHOD FOR THOSE POSSESSING NO FURNACE.

Obtain clear-cut impressions of cavity in quick-setting cement. When hard, place in ashes or similar plunger swager, using water-bag or plunger of unvulcanized rubber, place over impression a piece of No. 30 gold and swage. Over this place a piece of mat gold and reswage; a second piece of mat gold can be added and swaged again. The stiffened matrix can then be removed, held by the edge with pliers, filled with body, and the inlay baked over an ordinary Bunsen flame. If more gloss is desired, hold face down over flame for a moment. Peel off matrix, etch, and set. Where the color is of prime importance and I desire an exact reproduction of sample shades, I have had better success with Whiteley's and White's bodies. The possession of these, and others made from these, give a wide, adequate, and stable range of colors. Porcelain is equal to corners, if not a close square bite. No undercuts are used in inlays or cavities. A deepened cavity and etched inlay held in place when cemented, if possible, by a wedge for some time, and covered with paraffin to exclude moisture, seems to be equal to the demands. For proximal cavities a generous separation is essential to a perfect and unchanged matrix. I find I can handle $^1/_{1000}$ platinum as easily in most cases as the No. 30 gold when cavity is accessible, and the stiffer matrix is much more satisfactory. The color question, although the tooth is well matched to a sample shade, is a problem. The cement will change all inlays somewhat. A color somewhat lighter should be chosen. This problem can be solved to some extent by building in layers according to Dr. Reeves's method, with the yellow bases and overlying enamel color. In large proximal bicuspid fillings there seems to be a great tendency for the body to shrink and draw the platinum with it. I have found that a White's or similar tooth, crushed and powdered, and used as the foundation in fusing, and then burnished and finished with the enamel coloring, corrects the shrinkage. Used discriminately, the porcelain filling should be one of the available materials of every dentist.

The regular oxyphosphate filling has no place in my list of materials I use this extensively to cement, but not at all as a cement. I think that we do not begin to rely sufficiently upon the strength and range of usefulness of the adhesive qualities of cement. The oldest porcelain workers claim that redecay does not occur under a porcelain filling. Now, surely there is nothing in the nature of porcelain itself which has this retaining influence. It is the manner in which the filling is inserted, the plastic cement permeating every nook and corner under the inlay. If such is the case, why not by skilled manipulation, with our combined cement and metal fillings, our porcelain fillings, our gold or cast inlays or shell fillings, follow the plan of having all fillings inlays? As a filling-material I believe that cement causes the loss of more tooth-substance than it saves. It is short lived, unsatisfactory, unprofitable (for the patient, and this should be considered), unsightly, treacherous, and unstable. Very little dependence can be placed upon it. I now fill all children's anterior permanent teeth with gold or porcelain fillings in preference to cement. Gutta-percha is seldom used as a filling-material. To be sure, the pink gutta-percha will preserve the teeth and prevent decay, but this material is unstable, unclean after a season, it wedges the teeth apart, and causes recession of the gum and absorption of the alveolus in proximal cavities.

Until recently corners of anterior teeth, especially lowers with pulps and without, were a source of trouble to me because of inability to secure the necessary retention to withstand the force of occlusion. Now a gold inlay simplifies and makes a substantial restoration. It is, in fact, a miniature crown, and possesses the proportionate strength of such.

### METHOD OF CONSTRUCTION.

In a live tooth the cavity is prepared with no undercuts, the margins are smoothed, and a hole with No. 1 or No. 2 round bur is drilled in the seat at the cervical margin in the dentine parallel to the pulp-chamber to the depth of about one-sixty-fourth of an inch, or deeper if admissible. The palatal cutting edge of tooth is ground on a bevel and another hole drilled there between layers of enamel in dentine of a slight depth. One one-thousandth platinum is burnished to cavity as for an inlay, then platino-iridium wire, about 18 or 20 gauge, is pushed through the platinum and softened separately with platinum solder. I find the Turner blowpipe ideal for melting the platinum solder. When both pins are soldered, replace the matrix and thoroughly reburnish, using tape to hold platinum in contact with entire edge of cavity to prevent springing. Remove from cavity and engage one of pins in points of clamp pliers. Cover end of pliers and inside of matrix with whiting and water, dry out slowly and build up to proper contour with 20- or 22-carat solder, finish to size and cement in place. The solder for this work is prepared by fusing into globules with borax as flux, and applying these globules of solder when they are needed for fulness; pitting is obviated by so doing. In a pulpless tooth one large piece of wire inserted in canal is used.

CROWN- AND BRIDGE-WORK.

I do not insert a bridge unless there is a great need for it. I have no desire to mutilate a sound tooth as an abutment unless it is very imperative. I have practically abandoned gold crowns except in bridge-work. I use a hand-carved porcelain crown instead. Gold crowns as generally constructed are unhygienic and irritating to the gums, causing a recession of the same. This can be said of banded crowns. Formerly the Logan, and later the Davis crown were used most extensively. The Logan crown is good because of the strength of the porcelain and natural shape. The weak point is the post, which is prone to bend or stretch under the outward stress of mastication. A good joint at first on the palatal aspect becomes poor later because of bending of pin. Why the crown is not made with a platino-iridium post I fail to see. The time to secure an accurate fit is also a drawback with the Logan crown. Now a home-made crown on both posterior and anterior teeth is generally used. Carving teeth, which I always thought a difficult task and reserved for the gifted few, is not as hard as it seems, and can be accomplished with persistence by all.

METHOD OF CONSTRUCTION.

The root or roots are ground above the gum labially or buccally and palatally, so that the joint is above the gum to the fullest extent. Annealed platinum, about 36 to 40 gauge, burnished over end, and outline noted and platinum trimmed; the holes made for posts of platino-iridium wire from 13 to 15 gauge, canals drilled and posts inserted through platinum and soldered in correct relation with platinum solder. This plate and dowel is again carried to place and reburnished; impression and bite is then taken in modelling composition, and when the model is obtained the crown is built up in high-fusing body, using yellow as bulk and enamel color on outside. In molar teeth two or three posts are used. All-porcelain molar crowns are as strong as necessary, except in close bites, where little material can be used. In such cases the gold crown is imperative. In lower molars where the crown is all gone and the roots left standing independently, the all-porcelain crown can be used. The bridge across the gap can be spanned with platinum soldered to posts in the roots and crown built above. In case one or both are not on a level with the gum, the posts can be inserted in the roots of the canal, and built up with amalgam. A piece of platinum with holes for the posts is burnished over the ends of the roots; tubes are then made to slip over the posts and soldered to the plate, and the crown built up, covering the tubes. In the upper molar by this method I have produced from one-half to two-thirds of the palatal surface, together with the crown, when such recession and absorption had taken place. I find the Turner gasoline furnace equal to all demands for porcelain work. Teeth prone to become loose and remain in this condition should have a fixed permanent support. I believe that in such cases any method of ligaturing is only a temporizing contrivance. I have found a good splint to be made as follows: holes are drilled with a No. 2 bur through the affected and neighboring teeth above the pulp-chambers and as nearly parallel as possible. Gold wires are inserted in the holes and an impression taken. This is poured with wires in place, and a piece of pure gold is burnished over the model, soldered to the wires, stiffened with solder, and cemented in place. A rigid condition of these teeth is obtained, which condition, I believe, is largely instrumental in enabling the surrounding tissues to resume their normal state.

I have been as successful in bleaching teeth according to Dr. Head's method of saturating a tuft of cotton with the agent and applying this to the outside and inside of the tooth, and then applying a hot burnisher to the cotton to drive the active agent into the tooth, after which the enamel of tooth is ironed with a warm burnisher. Three per cent. peroxide, evaporated in an evaporating dish until as concentrated as desired, has proved as efficient as twenty-five per cent. pyrozone. Oxalic acid has also been used. It has seemed to me that there are some teeth other than those infiltrated with amalgam stain which are unaffected by bleaching agents.

The engine has been abandoned in cleansing the teeth except in removing deeply intrenched stains at the cervical margins of some teeth. The orange-wood stick and Ivory's port polisher as a carrier for pumice are used altogether.

I am more and more impressed with the conviction that the professional side of our work is exaggerated beyond measure. While the professional attitude should be maintained as far as the ethics are concerned in a free disclosure of methods, fair treatment of a brother practitioner, etc., the business side of dentistry should be more rigidly and systematically carried out. Few dentists receive fees which are commensurate with the best work. True dentistry is hard, skilful, tedious, nerve-exhausting labor, and should be rewarded accordingly. Our time is our capital, and in this respect it is different from most other professions. It is largely personal work, and cannot be relegated to others. I believe in charges for broken or unkept appointments when the time cannot be filled by another patient. The method of monthly bills for such part of work as is finished appeals to me and is appreciated by many patients. A big bill is harder to collect or even to pay.

### OBSERVATIONS AND REFLECTIONS.

It is better to discard mouth-mirrors after they have become hazy than to injure one's own mirrors. The latter cannot be replaced. Magnifying mirrors are used too frequently when plain mirrors would better be employed. Burs do lose their cutting edge and must have substitutes. An inverted cone bur will cut out fissure in a tooth about as rapidly as any other. In drilling out a post in a canal it is about as safe to proceed slowly, using up a few burs and cutting the pin itself rather than drill around it to loosen it. There is too much danger of perforation. Loose teeth are not always pyorrhœa subjects. Take note of the tooth opposing and lessen the strike by grinding. Proximal fillings which have been made to knuckle will be found to be separating from each other unless the occlusion and articulation upon the fillings are rectified. A direct squint at the cavity or progress of the work is often better than a reflected view in the glass, and reveals some defect. Cleanliness in person, instruments, and office equipment is keenly observed and rewarded.

It is a pretty good plan not to despoil a tooth until by experiment one is certain that the plan to be adopted is sure to be successful. Patients desire and are willing to pay for the best work if they can be convinced that it is the most satisfactory step to take, and they are to be supplied with honest work. Frank, plain talks with patients relative to the teeth are invaluable. Punctuality to appointments is essential on the part of both patient and operator. A dentist's time and advice are as valuable as that of a physician. I do not believe in the rapid operator in dentistry. It takes time to carefully, thoroughly, and wisely prepare a cavity; it takes time to insert and finish to the edges the plastic in the best manner; it takes time to condense fully the gold; and it takes time to finish the gold filling completely. It is not well to purchase every new device presented by the man who calls. The old one, to which you are accustomed, will work as well as the new. Do not jump at conclusions too quickly as to what is the best course to pursue. Think twice, and then think again, and then consult with another practitioner. Do every piece of work so that you are willing that your patient pass into another's hands; and such things happen sometimes, you know. Do not try to regulate the teeth without first making room for the new relations. Lack of room is the cause of condition as presented.

And now to come nearer home. I have wondered why it is that so few of our graduates attend the State and district dental meetings. Why is it that more of those connected with our school do not attend and become members of the National society? It has seemed to me that it behooved instructors and heads of departments in our school to get out of themselves and attend these National meetings and represent our school, and become infused with some new ideas other than those absorbed from this school. Local societies are well enough so far as they go, but they are circumscribed in their scope. If there is one weak point in our school system, it is that Harvard men impart only Harvard methods to our students. Now, there are constantly changing methods promulgated by men all over the dental world, and they should be demonstrated and tested here at the school.

How rarely do we find an Eastern man contributing to our dental magazines! In a recent expression of views on the value of the suction chamber, most every school had its contributor except the East. The lack of interest in other than local matters by our instructors and graduates, it seems to me, is a matter of regret, and one which should be corrected. It is a mistake to think that the best in dentistry revolves around this immediate vicinity. We should be elective in our study and practice, securing the best from all sources.

In these suggestions, together with more practical which have preceded in this cursory glance over my six years of practice, claim is made to originality in but few instances. If to any a kernel of help shall be found which can be utilized to lighten their labors, or help better their daily toil, the hope of the writer will be rewarded, as it was only to be helpful that consent was given to prepare this paper.

